# Effect of zinc oxide nanoparticles synthesized from *Carya illinoinensis* leaf extract on growth and antioxidant properties of mustard (*Brassica juncea*)

**DOI:** 10.3389/fpls.2023.1108186

**Published:** 2023-01-23

**Authors:** Addisie Geremew, Laura Carson, Selamawit Woldesenbet, Huichen Wang, Sheena Reeves, Nigel Brooks, Premkumar Saganti, Aruna Weerasooriya, Elisha Peace

**Affiliations:** ^1^ Cooperative Agricultural Research Center, Prairie View A&M University, Prairie View, TX, United States; ^2^ Department of Chemistry and Physics, College of Arts and Sciences, Prairie View A&M University, Prairie View, TX, United States; ^3^ Department of Chemical Engineering, College of Engineering, Prairie View A&M University, Prairie View, TX, United States

**Keywords:** zeta potential, flavonoids, net photosynthesis, chlorophyll content, macronutrients, micronutrients, reactive oxygen species (ROS)

## Abstract

**Background:**

The sustainability of crop production is impacted by climate change and land degradation, and the advanced application of nanotechnology is of paramount importance to overcome this challenge. The development of nanomaterials based on essential nutrients like zinc could serve as a basis for nanofertilizers and nanocomposite synthesis for broader agricultural applications and quality human nutrition. Therefore, this study aimed to synthesize zinc oxide nanoparticles (ZnO NPs) using pecan (Carya illinoinensis) leaf extract and investigate their effect on the growth, physiology, nutrient content, and antioxidant properties of mustard (Brassica juncea).

**Methods:**

The ZnO NPs were characterized by UV-Vis spectrophotometry, Dynamic Light Scattering (DLS), X-ray diffractometer (XRD), Scanning Electron Microscopy (SEM), and Fourier Transform Infra-Red Spectroscopy (FTIR). Mustard plants were subjected to different concentrations of ZnONPs (0, 20, 40, 60, 80, 100 and 200 mg L-1) during the vegetative growth stage.

**Results:**

The UV-Vis spectra of ZnO NPs revealed the absorption maxima at 362 nm and FTIR identified numerous functional groups that are responsible for capping and stabilizing ZnO NPs. DLS analysis presented monodispersed ZnO NPs of 84.5 nm size and highly negative zeta potential (-22.4 mV). Overall, the application of ZnO NPs enhanced the growth, chlorophyll content (by 53 %), relative water content (by 46 %), shoot biomass, membrane stability (by 54 %) and net photosynthesis significantly in a dose-dependent manner. In addition, the supplement of the ZnO NPs augmented K, Fe, Zn and flavonoid contents as well as overcome the effect of reactive oxygen species by increasing antioxidant capacity in mustard leaves up to 97 %.

**Conclusions:**

In conclusion, ZnO NPs can be potentially used as a plant growth stimulant and as a novel soil amendment for enhancing crop yields. Besides, the biofortification of B. juncea plants with ZnO NPs helps to improve the nutritional quality of the crop and perhaps potentiates its pharmaceutical effects.

## 1 Introduction

Globally, the sustainability of crop production is impacted by several factors including climate change and land degradation (Webb et al., 2017; Jiang et al., 2021). To maintain sustainable agriculture and food production, the advanced application of nanotechnology is of paramount importance (Fraceto et al., 2016; Wang et al., 2018; Neme et al., 2021). Its application improves agricultural production by reducing losses and enhancing the efficiency of inputs (Manjunatha et al., 2016; Shang et al., 2019; Neme et al., 2021) and crop yields and productivity (Noohpisheh et al., 2021). Nanoparticles are nanomaterials with peculiar physicochemical characteristics including enhanced reactivity, typical surface structure, and high surface-to-volume ratio (Noohpisheh et al., 2021; Badawy et al., 2021). Owing to these attributes, NPs are used as nanofertilizers (Jiang et al., 2021; Noohpisheh et al., 2021; Awan et al., 2021) and reduce nutrient deficiency (Etienne et al., 2018). Thus, supplying controlled and targeted mineral nutrient release to plants (Salama et al., 2019) and then resulting in increased crop growth and development (Wang et al., 2018; Rajput et al., 2021; Srivastava et al., 2021). However, recent studies have revealed that NPs may show both positive or negative impact on plants which mainly depends on the chemical structure, size, reactivity, and dose (Elizabeth et al., 2017; Salama et al., 2019) that vary according to plant species (Rastogi et al., 2017; Regni et al., 2022).

Plants face uninterrupted fluxes of environmental conditions and are frequently subjected to associated abiotic stresses such as drought, salinity, heavy metals, waterlogging, extreme temperatures, and oxygen deprivation, which influence plant growth, and development, ultimately impacting yield and quality (Kapoor et al., 2020; Zulfiqar and Ashraf, 2022a). Plants exposed to abiotic stress, singularly or in combination, produce excess reactive oxygen species (ROS) which leads to oxidative stress and impaired redox homeostasis. (Noctor et al., 2018; Hasanuzzaman et al., 2020; Zulfiqar and Ashraf, 2021). In addition to their negative impact, ROS play a significant function as secondary messengers or signaling molecules in different cellular mechanisms to increase tolerance against various abiotic stresses (Singh et al., 2019; Hasanuzzaman et al., 2020), specifically during the acclimation processes (Antoniou et al., 2016). The balance between ROS generation and the antioxidant defense system protects plants from the impact of stress. However, to withstand oxidative stress caused by ROS over-accumulation, plants activate their endogenous antioxidant defense mechanisms, either enzymatically or non-enzymatically (Hasanuzzaman et al., 2020; Lukacova et al., 2021). The enzymatic antioxidants defense involves the production of diverse enzymes such as superoxide dismutase, catalase, ascorbate peroxidase, glutathione reductase, monodehydroascorbate reductase, dehydroascorbate reductase, glutathione peroxidase, glutathione, peroxiredoxins, ferritin, thioredoxins and glutaredoxin (Gill and Tuteja, 2010; Kaur et al., 2019). Whereas the nonenzymatic antioxidant mechanism encompasses the production of ascorbic acid, glutathione, phenolic acids, alkaloids, flavonoids, carotenoids, alpha-tocopherol, nonprotein amino acids, etc. (Gill and Tuteja, 2010; Zulfiqar and Ashraf, 2022a). To overcome oxidative stress, plants have also employed osmolyte accumulation like proline as endogenous strategies (Zulfiqar and Ashraf, 2022b). Proline can scavenge free radicals generated through osmoprotection, osmoregulation, ROS quenching, metal chelation, and buffering of cellular redox potential of plants under various stressors (Zulfiqar and Ashraf, 2022b). Zulfiqar et al. (2019) have also highlighted the role of osmoprotectants such as amino acids, polyamines, quaternary ammonium compounds and sugars in mitigating the negative effect of abiotic stress by scavenging ROS, acting as metabolic signals and stabilizing cellular structures and enzymes. Moreover, a recent study has also shown that foliar application of ascorbic acid mitigates the adverse effects of salinity on lettuce *(Lactuca sativa*) by reducing oxidative injury (Naz et al., 2022). Different plants have varied capacities to tolerate oxidative stress that depends on the ability of their antioxidant machinery. Research towards increasing the antioxidant defense in plants is vital. These days, the applications of metallic NPs are thought to be sound solutions for ameliorating different stresses through increasing antioxidant enzymes (Faizan and Hayat, 2019; Hasanuzzaman et al., 2020; Alabdallah and Alzahrani, 2020).

Zinc is an essential micronutrient required for a broader range of plants’ key functions such as improving water use efficiency, photosynthesis, protein synthesis, regulation of reactive oxygen species, antioxidant function, maintenance of membranes integrity, growth regulation, and gene expression (Bagci et al., 2007; Wang et al., 2018; Noohpisheh et al., 2021). In addition to these functions, from the consumer perspective considering the basic benefits of Zn in human health, the bio-fortification of crops with such essential nutrients through the application of nanomaterials has recently gained attention (Iziy et al., 2019; Salama et al., 2019). To comply with the zinc demands of plants, ZnO NPs have been reported as the smartest delivery tools that substitute the zinc conventional fertilizer and increase the availability of Zn for plants (Rajput et al., 2021; Awan et al., 2021).

ZnO NPs application has enhanced plants growth, photosynthesis and development of corn, onion, tomato, olive, capsicum, cucumber, wheat, and zucchini (Stampoulis et al., 2009; Zhao et al., 2013; Munir et al., 2018; Wang et al., 2018; Neto et al., 2020; Awan et al., 2021; Regni et al., 2022) in a dose-dependent manner. Nevertheless, the response of plants to ZnO NPs application is a function of genotypes, the stage of the plant, and the concentration of nanoparticles provided (Salama et al., 2019). On the other hand, the application of chemically synthesized ZnO NPs has been criticized compared to the biosynthesized counters (Rai et al., 2018; Jamkhande et al., 2019). In fact, the latter is regarded as environmentally friendly, safer and potentially more efficiently obtained using plant extracts (Jamkhande et al., 2019; Jangannanavar et al., 2021; Regni et al., 2022). However, the use of biologically synthesized ZnO NPs as nanofertilizers to enhance zinc content and improve morpho-physiological traits and antioxidant properties of leafy vegetables at early vegetative stages is limited (Iziy et al., 2019; Salama et al., 2019; Regni et al., 2022).

Mustard (*Brassica juncea* (L.) Czern) belongs to the Brassicaceae family. The green vegetables and seeds of mustard are economically valuable and widely consumed by humans due to their astonishing provision of several health-promoting metabolites and nutrients (Sharma et al., 2018; Majdoub et al., 2020; Geremew et al., 2021). Overall, studies have shown that high consumption of mustard is linked with the prevention of several cancers (Kwak et al., 2016), antioxidant activities and inhibition of fat increase (Kim et al., 2003). Despite these economic and health benefits, poor soil fertility during its vital stages such as seed germination, growth, flowering, and pod filling severely impacts crop yield (Geremew et al., 2021). Owing to these dietary and economic values, mustard plants need special interest to boost their production and their nutritional value under limited soil fertility.

To fulfill this ever-increasing need for nutrients, only soil is not adequate, the micronutrients and macronutrients should be supplemented in the soil in very small quantities (Iris et al., 2018) in the form of nanoparticles (Iziy et al., 2019). Therefore, in the present study, we synthesized ZnO NPs using pecan (*Carya illinoinensis*) leaf extract and investigated their effect on the growth, physiology, nutrient content, and antioxidant properties of mustard (*B. juncea*). Specifically, we asked the following questions: (i) Do ZnO NPs affect the morpho-physiological traits of mustard plants? (ii) Do ZnO NPs enhance the macro- and micronutrient contents of mustard leaves in a concentration-dependent manner? (iii) How ZnO NPs affect the antioxidant properties of mustard plants?

## 2 Materials and methods

### 2.1 Chemicals used

All chemicals used were of analytical grades. Zinc nitrate hexahydrate (99%), methanol, DPPH (99%), acetone, ethanol, ascorbic acid (99%), sodium hydroxide (99%), aluminum trichloride, potassium acetate and quercetin were purchased from Sigma Aldrich (Burlington, MA). MitoSOX™ Mitochondrial Superoxide Indicators (Invitrogen™, M36008), and Propidium iodide in 1mg/ml aqueous solution (Thermo Scientific™, J66584.AB) were used.

### 2.2 Plant sample collection and extraction

Green leaves of pecan (*Carya illinoinensis* (Wangenh). K. Koch) were collected from the Bill and Vara Daniel Farm and Ranch located at Prairie View A&M University (PVAMU) and the sample was identified and the voucher specimen was stored at the Cooperative Agricultural Research Center (CARC) at Prairie View A&M University. The collected leaves were cleaned by rinsing in distilled water several times to remove debris. Subsequently, the leaves were freeze-dried using BenchTop Pro with Omnitronics™ freeze dryer (BTP-8ZL00W, SP Scientific, PA, USA) and grounded manually using mortar and pestle. Next, the fine leave powder (15 g) was added to 400 mL deionized water and shaken with an orbital shaker (IKA Basic Variable-Speed Digital Orbital Shaker, model, 115 V) at 200 rpm at 30°C for 48 hrs. The extract solution was filtered using Stericup^®^ Quick Release Vacuum driven disposable filter (integrated with Millipore Express^®^ Plus 0.22 µm PES System (Sigma Aldrich). The filtrate was kept at 4°C pending the synthesis of ZnO NPs.

### 2.3 Biosynthesis of ZnO nanoparticles

ZnO NPs were synthesized using a modified method suggested by Jayachandran et al. (2021). Zinc nitrate hexahydrate (Zn (NO_3_)_2_.6H_2_O) was used as a precursor for the synthesis of the ZnO NPs. Ninety (90) mL of 1 mM Zn (NO_3_)_2_.6H_2_O was poured into 10 mL of pecan leaf extract in a 200 mL flask. The mixture was stirred at 65°C for 25 min until light yellow colloidal suspension formed. This colloidal suspension was further centrifuged at 10,000 RPM for 10 min twice. The pellet was retained and washed with ethanol to remove the remaining organic matter and centrifuged again at the same speed. The pellet was completely dried and calcinated at 600°C for 2 hrs under furnace (Thermo Fisher). We further removed organic matter (ash) from calcination by washing the powder in ethanol and centrifuging at 10,000 RPM for 10 min. Pending the characterization of the nanoparticles, the collected pellet was dried, crushed and stored in dark glass under a desiccator.

### 2.4 Characterization of ZnO NPs

The sample of ZnO NPs (6 mg) was dissolved in 10 mL of double distilled water for characterization. To measure the optical parameters, the synthesized ZnO NPs were dispersed in deionized water. The absorption spectrum of the ZnO NPs was determined using UV-VIS Spectrophotometer (SpectraMax^®^ PLUS 384, England) in a spectrum range between 200-800 nm. Deionized water was used as a reference. The surface chemistry of functional groups and biomolecules attached to the ZnO NPs was analyzed by FTIR spectrometer (JASCO/FTIR-6300, Japan) with a resolution of 4 cm^-1^ at a frequency of 4,000-500 cm^-1^. The particle size distribution and zeta potential of the samples were obtained through dynamic light scattering (DLS) procedure operated using Litesizer™ 500 (Anton Paar, Austria) coupled with a 10 mW He-Ne laser (633 nm) running at an angle of 90° and temperature of 25°C. Water was used as a dispersant to measure the zeta potential. In addition, the evaluation of the morphology of ZnO NPs was performed using scanning electron microscopy (SEM) coupled with an energy-dispersive x-ray spectroscopy (EDX) system (JOEL JSM-6010LA, Japan). The EDX spectrometry particularly was run to identify and quantify the elemental composition of the nanoparticles. X-ray diffractometer (XRD-7000, Shimadzu, Japan) run at 40 kV and 30 mA was used to examine the surface morphology, size and crystalline nature of ZnO NPs. The diffraction pattern was recorded by CuKα radiation with a wavelength of λ = 1.541 Å. The scanning was carried out in 2θ value range of 10° to 80° at 0.02 min^-1^ and 1 second time constant. Scherrer’s equation was used to compute the average crystalline size of synthesized ZnO NPs as:


(1) Dp=0.9λ/βCosθ

Where Dp represents the average crystallite size, λ stands for the wavelength (1.5406 Å for Cu Kα), β designates the full width at half maximum (FWHM) of main intensity peak after subtraction of the equipment broadening and θ is used as a diffraction angle in radians.

### 2.5 Plant material and growth conditions

The experiment was carried out in a plant growth chamber at CARC, PVAMU, Texas, USA during the Summer of 2022. Seeds of the Indian mustard (*Brassica juncea* (L.) Czern) were acquired from Twilley seed company (Hodges, SC, USA). Ten seeds were sown in plastic pots (8-inch size) containing 2 kg of sieved clay soil, electrical conductivity, and pH of 0.7925 dS m^−1^, and 7.65, respectively. After germination, seven seedlings were thinned to ensure that every pot comprised three plants of the same vigor. Pots with mustard seedlings thoroughly received 200 mL suspensions of 0 (deionized water), 20, 40, 60, 80, 100, and 200 mg L^-1^ ZnO NPs directly on the soil after 20 and 40 days of germination. A completely randomized design with four replications of each treatment was applied. All pots were irrigated with distilled water twice a week. The time and list of measurements carried out are summarized in **Supporting Figure 1**.

### 2.6 Morpho-physiological variables

#### 2.6.1 Measurement of growth

Plant height was measured from the stem base of mustard plant to the tips of its shoot using a meter 45 days after treatment. Forty-five days after germination leaf area (LA) was determined from measurements of leaf length and width using the equation:


(2) LA=0.72 x length x width

where 0.72 is the correction factor for leaf area in mustard plants adopted from Ramil and Sulaiman (2021). Individual plants that were separated into roots and shoots and leaves and were dried in an oven at 70°C until their constant weight acquired 60 days after germination. These dried weights were their respective biomass values (below-ground biomass and above ground biomass, respectively).

#### 2.6.2 Membrane stability index

Membrane stability index (MSI) was determined 45 days after germination following the method suggested by Sairam (1994). One gram of sample containing 5 leaf portions, 4 cm long each, was immersed in a test tube with 15 mL of distilled water. The submersed samples were incubated for 24 hrs at 20 ^°^C. Subsequently, the electrical conductivity of the water (C1) was measured using conductivity meter HI198129 (Hanna Instruments Inc., Woonsocket, Rhode Island). Then, we boiled the samples at 100 ^°^C for 10 min and conductance was noted (C2). Membrane stability was computed as:


(3) $$\text{MSI=}\left(1-\left(\frac{c1}{c2}\right)\right)*100$$

#### 2.6.3 Relative water content

Relative water content (RWC) was quantified by applying the method of Barrs and Weatherley (1962). Healthy and fully expanded leaves were collected from individual plants 45 days after germination and cut into 6 x 6 mm^2^ discs. Fresh weight (FW) of these discs was measured and then immersed in distilled water for 12 hrs. Afterward, the exterior of the discs was dried using tissue paper and then turgid weight (SW) was recorded. Thereafter, the discs were dried in an oven at 70°C for 24 hrs and dry weight (DW) was recorded. RWC in percent was then calculated as:


(4) $$RWC=\left(\frac{FW-DW}{SW-DW}\right)\;X\;100$$

#### 2.6.4 Photosynthetic pigments content

The overall chlorophyll contents in the intact leaves of mustard plants were measured using Chlorophyll meter, SPAD-502 (Minolta Co., Ltd., Osaka, Japan). The SPAD values were taken at the leaf lamina and towards the tip. The observations were made early in the morning between 10:00 and 11.00 a.m. To further analyze the various components of photosynthetic pigments, 200 mg mustard leaves of ZnO NPs treated and untreated plants were extracted in 20 mL of chilled acetone: ethanol (1:1, v/v) and kept in dark for 24 hrs under room temperature. This extract was centrifuged at 8,000 RPM for 10 min, and the supernatant was collected. After centrifugation, the absorbance of the supernatant was taken at 663, 645 and 480 nm. Chlorophyll a, chlorophyll b, and carotenoid contents were estimated in mg, per g of fresh weight following methods by Ulhassan et al. (2019).

#### 2.6.5 Photosynthetic pigments content

Net photosynthetic rate (Pn), leaf stomatal conductance (gs), intercellular CO_2_ concentration (Ci), and transpiration rate (E) of the second young and fully expanded three mustard leaves were recorded using a portable photosynthesis system (Li-Cor 6400XT, Lincoln, NE, USA). The measurements were conducted under the conditions of photosynthetically active radiation of 1000 μmol m^-2^ s^-1^, an ambient CO_2_ concentration of 360 ± 10 μmol mol^-1^, air temperature of 22°C, and relative humidity of 50%. Three leaves per pot were measured two times per leaf.

### 2.7 Determination of macro- and micronutrients

Young leaves of the mustard plant were obtained for macro- and micronutrient analysis after 60 days of treatment application. The leaves were freeze-dried using BenchTop Pro with Omnitronics™ freeze dryer (BTP-8ZL00W, SP Scientific, PA, USA) for 12 hrs to constant weight and were ground manually using mortar and pestle. For microwave digestion, about 250 mg of each mustard leaves sample were directly placed into a microwave closed vessel. Then, 2 mL of 30% H_2_O_2_ and 7.0 mL of 65% (m/m) HNO_3_ solutions were poured into each vessel. Digestion was run with a high-pressure microwave oven (Milestone Ethos UP 1600, Sorisole, Italy) at a frequency of 2450 Hz. The digested samples were filtered through a 0.45 µm nylon membrane (Millipore Sigma™ Millex™-GP Sterile Syringe Filters, Burlington, Massachusetts). The concentration of P, K, Ca, Cu, Mg, Fe, Mn, Na, and Zn in each sample were analyzed using radial view of Inductive Coupled Plasma Optical Emission Spectrometer (ICP-OES, Agilent ICP-5100) equipped with Agilent SP4 autosampler.

### 2.8 SEM analysis of Zn accumulation in the leaves

The leaves obtained after 60 days after treatment were also subjected to SEM to detect the accumulation of ZnO NPs in the leaf. Briefly, the freeze-dried leaf samples (24 hrs at 50°C) were sectioned and sputter-coated with carbon and affixed on an aluminum stub. Then the samples were then imaged with SEM with EDX on a JEOL JSM-6610 (Oxford Instruments). Percent zinc and other nutrients were calculated.

### 2.9 Reactive oxygen species analysis

The leaves were first cut in 1 in square and dissected into a small slice under a stereomicrosopy (Motic SMZ-168 Series; Motic, Hong-Kong, China). The slide was put on glass slide. For superoxide analysis, samples were incubated in 5 μM MitoSOX Red in darkness for 30 min at room temperature. After three washes, the plant tissues were immediately imaged with a Leica SP8 confocal laser-scanning microscope (SP8) with the excitation/emission at 405/516-580 nm (Leica Microsystems, Wetzlar, Germany) equipped with an HC PL CS2 20×/0.75.

### 2.10 Total flavonoids content in the leaves

The total flavonoid content in *B. juncea* leaves was determined by the colorimetric method of aluminum trichloride as described by Chang et al. (2002). A 0.5 mL aliquot of the ethanol extract of *B. juncea* leaves was mixed with 2.8 mL of water, 1.5 mL of 95% ethanol, 0.1 mL of 10% aluminum trichloride and 0.1 mL of potassium acetate (1 M). The mixture was vortexed and allowed to stand for 30 min. The absorbance was measured with a UV-Vis spectrophotometer (SpectraMax^®^ PLUS 384) at 424 nm. Quercetin was used as a standard solution. The total flavonoid content was expressed as quercetin equivalents (mg QE g^-1^ dry leaf).

### 2.11 Antioxidant activity

To assess the effect of ZnO NPs treatment on the antioxidant potentials of mustard plants the radical 2, 2-diphenyl-1-picrylhydrazyl (DPPH) assay was performed with a modification of Choi et al. (2002). Fifty (50) μL of each ZnO NPs treated mustard plant leaf methanolic extracts were mixed with DPPH radical solution in methanol (0.1 mM, 150 μL) in 150 mL flasks. Each flask was covered with aluminum foil and incubated at room temperature in the dark for 30 min. Then the absorbance was recorded at 517 nm using a UV-Vis spectrophotometer (SpectraMax^®^ PLUS 384). DPPH methanol reagent without the leave extract was used as control and percentage radical scavenging activity was determined as:


(5) $$\text{Radical Scanging activity}(\%)=\left(\frac{{\text{A}}_{0}{\text{-A}}_{1}}{{\text{A}}_{0}}\right)\;*\;100$$

where A_0_ and A_1_ represent the OD of the ascorbic acid and the ZnO NPs treated leaf extracts, respectively.

### 2.12 Statistical analysis

The experiment was carried out in four replicates and the data was subjected to one-way analysis of variance (ANOVA) using R v 3.5 (http://www.R-project.org) and the agricolae package (Mendiburu, 2013) and expressed as mean values ± standard error. Tukey multiple comparison test (significance level 5%) was used to calculate the differences between each concentration level of ZnO NPs. Sigma Plot Software (Version 14.5) was used for graphical presentation.

## 3 Results and discussion

### 3.1 Zinc oxide nanoparticles characteristics

**Figure 1:**
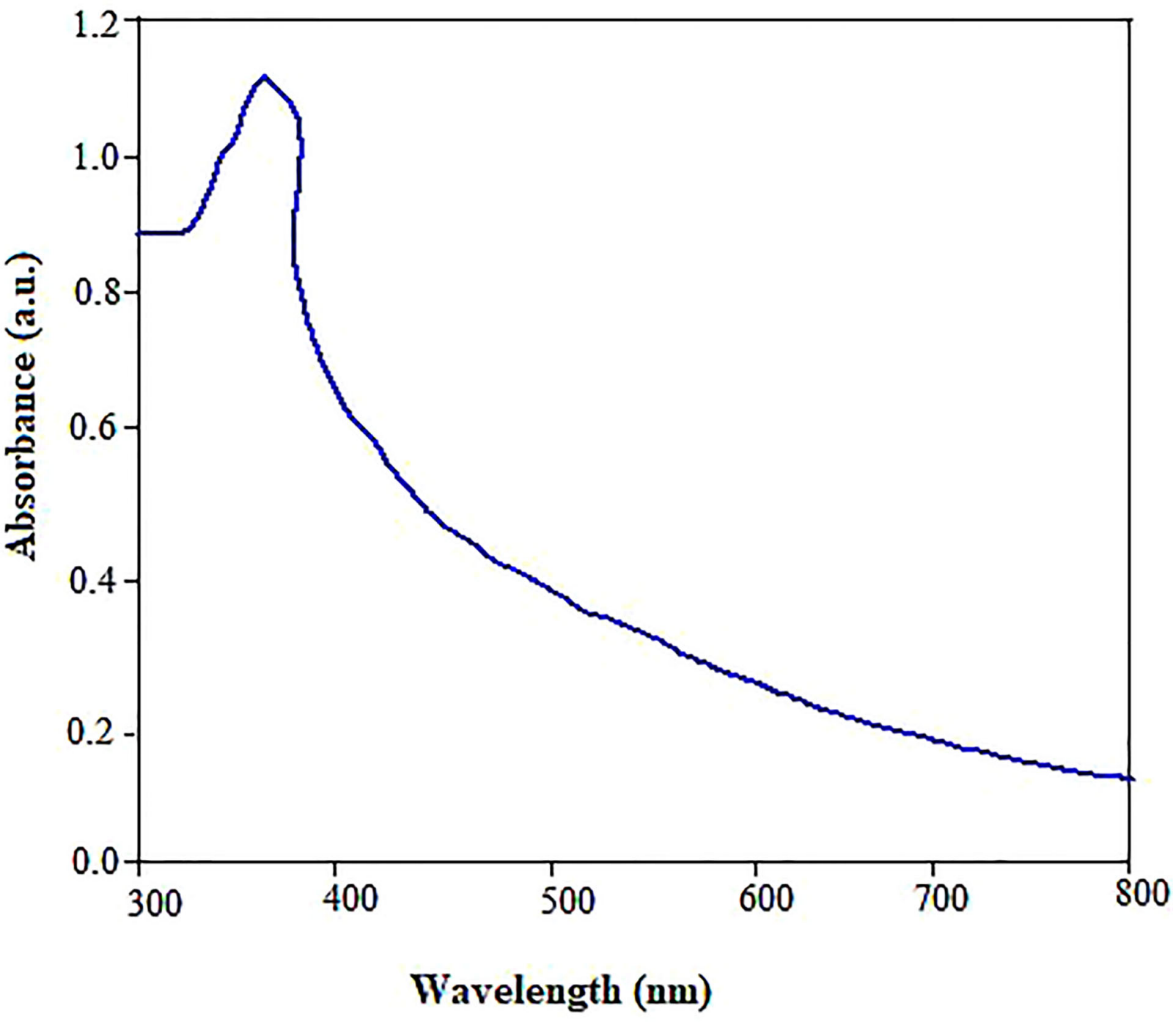
UV-Vis absorbance spectra of ZnO NPs synthesized using pecan leaf extracts.

The focus of the present study was to test the hypothesis if the biologically synthesized ZnO NPs using pecan (*C. illinoinensis*) leaf extract could be used as an environmentally friendly alternative nanofertilizer or growth stimulating agent for enhanced physiological performance, nutrient content, and antioxidant properties of mustard (*B. juncea*). While the reaction between Zn (NO_3_)_2_.6H_2_O and pecan extract progressed, the color transformation of the reaction mixture from light green to creamy yellow after the incubation period indicated the biosynthesis of ZnO NPs. The optical absorption band of ZnO NPs was analyzed by UV-vis spectrometer to monitor and confirm the formation and stability of the nanoparticles (**Figure 1**). The absorption spectra of the green synthesized ZnO NPs showed a maximum optical absorption peak at 362 nm. The peak recorded between 320 and 380 nm could also be associated with phenolic compounds (Ozsoy et al., 2008), which are involved in the reduction and stabilization of ZnO NPs. The peak pattern observed matches the typical characteristic of ZnO NPs (Sharmila et al., 2018). Despite the surface plasmon resistance (SPR) is a function of the diameter, shape and size distribution of ZnO NPs (Narayana et al., 2020) other studies also reported plasmon peak appears between 320 to 380 nm (Zare et al., 2017; Salama et al., 2019). Similarly, free electrons present SPR.

**Figure 2:**
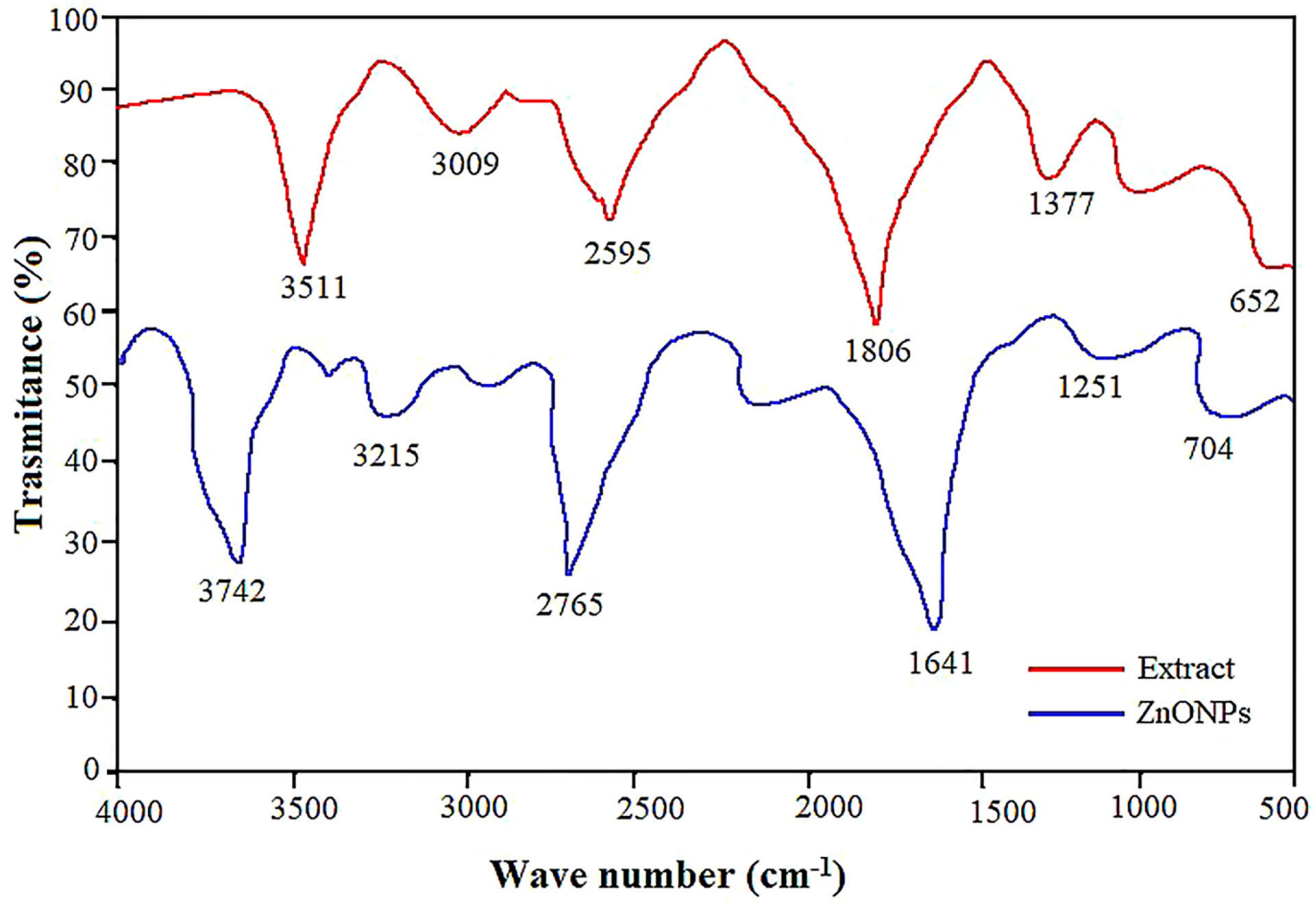
Comparison of the FTIR spectra of the pecan leaf extract and ZnO NPs. Each peak in the ZnO NPs indicates the functional group of the phytochemical involved in nanoparticle synthesis.

FTIR analysis was carried out to analyze the composition, the nature of the functional groups, and the purity as well as identify the potential mechanism of the ZnO NPs synthesis. The observed FTIR spectrum for ZnO NPs showed peaks at 3742, 3215, 2765, 1641, 1251 and 704 cm^-1^ (**Figure 2**). Pecan leaf extract exhibited various functional group stretches between 3511 cm^-1^ and 652 cm^-1^. However, the peaks detected in the extract were observed to shift in the ZnO NPs inferring the role of different functional groups in the bioreduction and stabilization of ZnO NPs (Geremew et al., 2022). The ZnO NPs exhibited strong transmittance spectra at 3742 cm^-1^ representing the N-H stretch strong amines group (Umamaheswari et al., 2021; Geremew et al., 2022), 3215 cm^-1^ associated with the hydroxyl group stretching vibration of phenol or flavonoids (Adil et al., 2019). In addition, the 1641 cm^-1^, 2765 cm^-1^ and 1251 cm^- 1^ bands linked to a carbonyl group (C=O) (Rastogi et al., 2011), alkyl methylene group (C=H) and C-O group bonded with strong alcohols, respectively. Furthermore, 1377 cm^-1^ representing C=C strong stretch assigned to aromatic group and 712 cm^-1^ for C-H bond assigned to strong mono-substituted aromatic benzene group (Geremew et al., 2022).

**Figure 3:**
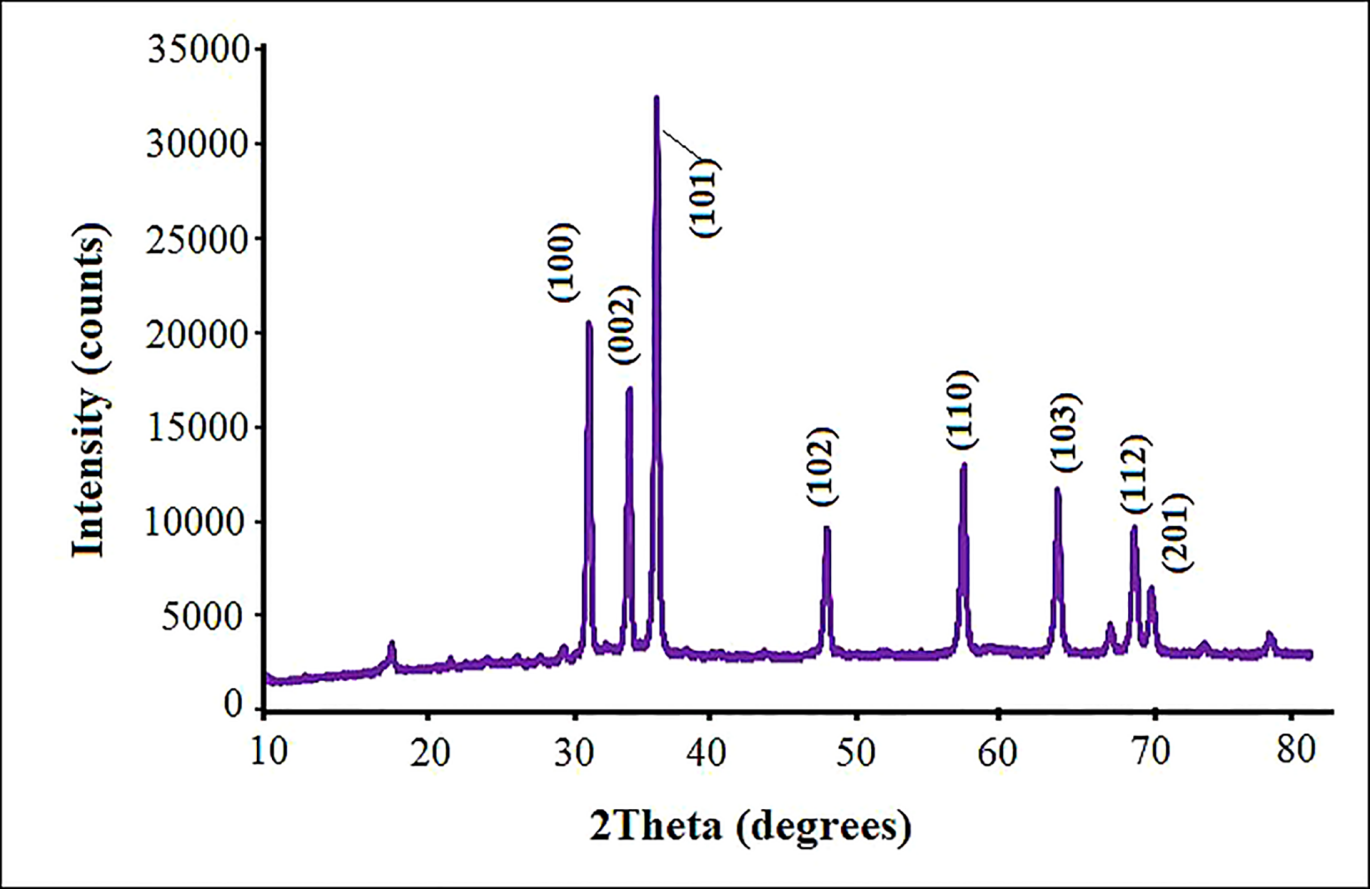
X-ray diffractometer patterns for biosynthesized ZnO NPs using pecan leaf extract.

The purity, crystalline nature and size of the fabricated ZnO NPs were measured by XRD analysis in the scanning angle (2θ). According to the XRD pattern, ZnO NPs displayed sharp peaks at 2θ values of 31.61^°^, 33.92^°^, 36.74^°^, 47.61^°^, 56.67^°^, 62.95^°^, 68.03^°^, and 71.65^°^ (**Figure 3**). These peaks, respectively, correspond to the diffraction planes 100, 002, 101, 102, 110, 103, 112 and 201, which confirmed the hexagonal wurtzite ZnO NPs structure (Bala et al., 2015). These values of ZnO NPs were in good agreement with the standard value (JCPDS No. 36-1415) (Jabeen et al., 2017), reflecting the phase purity of ZnO NPs. All peaks were appropriated to the ZnO NPs structure reported by Chen et al. (2008) and by Archana et al. (2016). The crystallite size was about 53.2 nm as computed by the Scherrer formula.

**Figure 4:**
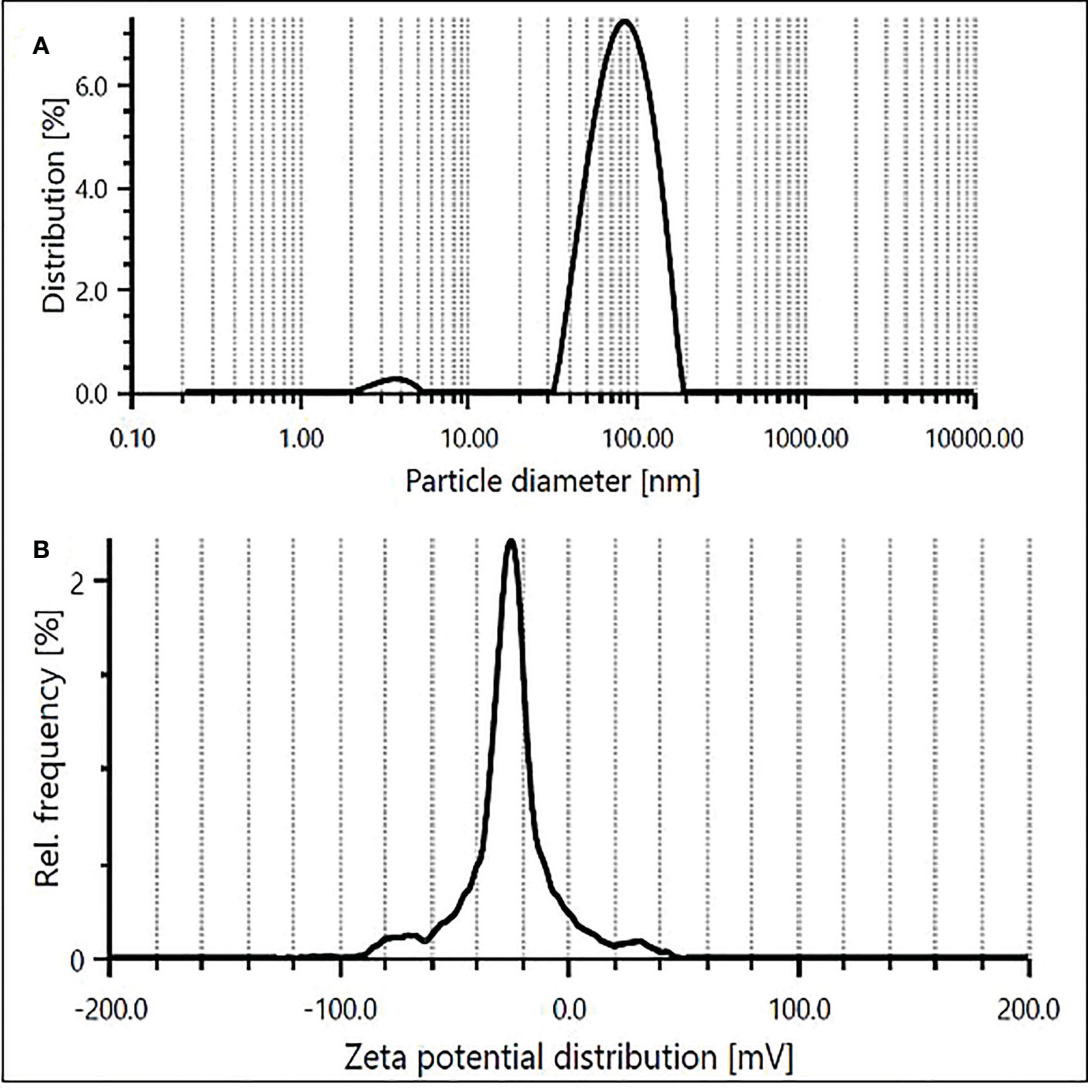
Size distribution **(A)** and zeta potential **(B)** of ZnO NPs obtained using aqueous extracts of pecan leaves.

DLS technique was applied to determine the hydrodynamic diameter of ZnO NPs in the aqueous suspension. The particle diameter distribution showed a stable colloidal suspension of ZnO NPs with a mean size of 84.5 nm (**Figure 4A**). This size is relatively larger than the theoretical size of the ZnO NPs computed using XRD, indicating the agglomeration of the nanoparticles attributable to the presence of ions and phytochemicals such as capping and stabilizing agents attached to surfaces of ZnO NPs in aqueous suspension (Jamdagni et al., 2018). However, the polydispersity index (PDI) of 0.37 in the present study exhibited monodispersion and homogeneity of the NPs in the medium. As a proxy of the stability of biologically synthesized ZnO NPs, negative zeta potential of -22.4 mV was recorded (**Figure 4B**), ascertaining the efficacy of phytochemicals in pecan leaf extract as capping agents in the stabilization of the particles. The negative zeta potential value of ZnO NPs could be ascribed to negatively charged capping agents attached to the surface of nanoparticles (Wei et al., 2020). Conventionally, the zeta potential values between + 25 and − 25 mV mark a stable suspension of nanoparticles (Kuznetsova and Rempela, 2015; Murali et al., 2017), which validates the high stability (−22.4 mV) of synthesized ZnO NPs colloidal suspension in this study.

**Figure 5:**
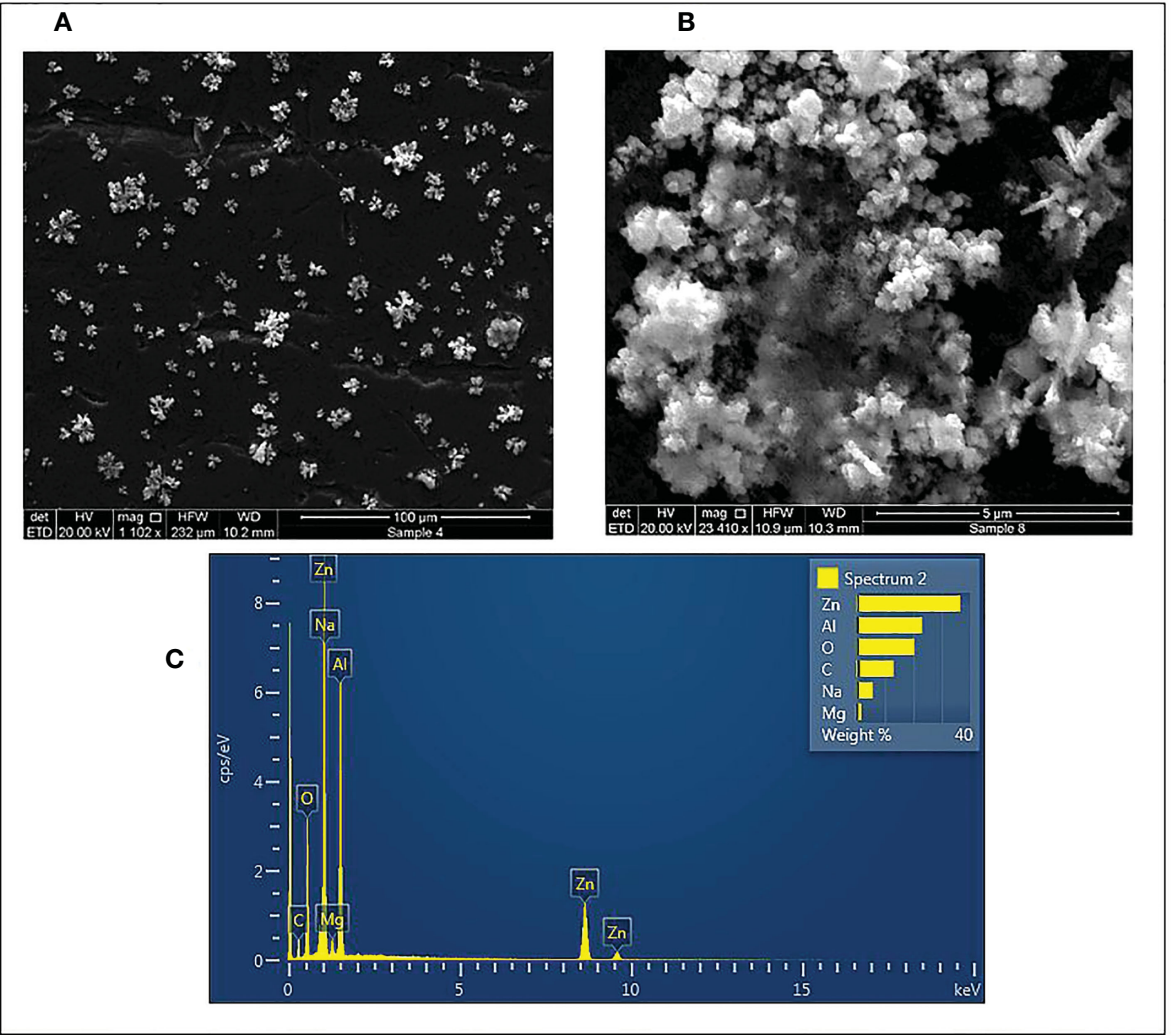
SEM micrographs 1102X **(A)** and 2340X **(B)** and, EDX spectra **(C)** of synthesized ZnO NPs using pecan leaf extract as a reducing agent. The inset bar plot of the EDX showed the percent weight of the proportion of Zn, Al, O, C, Na and Mg.

Additionally, the shape and surface morphology of pecan leaf extract-mediated ZnO NPs were assessed by SEM. SEM images of ZnO NPs at different magnifications are shown in **Figures 5A, B**. The SEM analysis depicted star-shaped ZnO NPs with slight agglomeration and regular morphology. The elemental composition and chemical purity of ZnO NPs were studied by EDX Spectroscopy. EDX analysis revealed that zinc is the primary constituent (45%) with strong peaks at 1, 8.6 and 9.6 keV due to the SPR effect of ZnO NPs (**Figure 5C**). In addition, carbon, oxygen, sodium and magnesium were detected as elemental components that might be associated with the pecan leaf extract used for the synthesis of the nanoparticles. Aluminum was detected as a major element due to the grid used.

### 3.2 Effect of ZnO NPs on mustard plant growth

Understanding the effect of nanoparticles synthesized on plant growth and development is a significant metric of toxicity and evaluation tool prior to agricultural application at a large scale. Plant growth traits such as height, leaf area and biomass are extensively used as biomarkers for phytotoxicity (Ali et al., 2015). In the present study *B. juncea* plants exposed to the different concentrations of ZnO NPs (20, 40, 60, 80, 100 or 200 mg L^-1^), enhanced the values for all the growth traits such as an increase in height, leaf area, and shoot dry weight compared to the control (distilled water) at early vegetative stage (**Table 1**). Phytotoxicity test on mustard percent seed germination is provided in **Supplementary Figures 2 A-C**. Maximum leaf area (85.5 cm^2^), height (77.4 cm), and shoot dry weight (46.5 g) were found to be 52%, 45% and 78%, respectively, higher than the control (40.91 cm^2^, 42.91 cm and 10.32 g) in plants treated with 200 mg L^_1^ ZnO NPs (**Table 1**). At 100 and 200 mg L^-1^ these traits were significantly higher than plants in the control (P < 0.05). However, a significant reduction in root dry weight at higher concentrations (100 and 200 mg L^-1^ ZnO NPs) was recorded. Though the function of nanoparticles depends on their properties and methods of synthesis (Liu et al., 2020), with the application of chemically synthesized ZnO NPs Rao and Shekhawat (2014) reported a dose-dependent significant reduction in shoot length of *B. juncea* under higher concentrations (1000 and 1500 mg L^-1^). Similarly, other research showed that the addition of ZnO NPs enhanced the growth of *Lolium perenne* (Lin and Xing, 2007), *Allium cepa* (Laware and Raskar, 2014), *Olea europaea* (Micheli et al., 2018), *Brassica nigra* (Zafar et al., 2016), *Cicer arietinum* (Burman et al., 2013), *Capsicum annuum* (Deore et al., 2010), *Capsicum annuum* (Datir et al., 2012), *Zea mays* (Neto et al., 2020), *Gossypium hirsutum* (Venkatachalam et al., 2017), *Triticum aestivum* (Munir et al., 2018), *Brassica oleracea* (Awan et al., 2021) and *Cucumis sativus* (Zaho et al., 2013). In contrast, Chen et al. (2018) and Wang et al. (2018) found that growth of the *Oryza sativa* and *Solanum lycopersicum* plants, respectively, significantly decreased at 100 and 200 ppm of ZnO NPs treatment. This suggests that the effect of ZnO NPs on plant growth may strongly depend on the plant species (García-Gómez et al., 2017) and its dose.

**Table 1:** Effects of ZnO NPs application on morpho-physiological traits of *B. juncea*.

Treatment (mg L^-1^)	Height (cm)	LAI (cm^2^)	RWC (%)	MSI (%)	SPAD	SDW (g)	RDW (g)	Pn (µmol (CO_2_) m^-2^s^-1^)	E (mmol m^−2^ s^−1)^	gs (mmol m^−2^ s^−1)^	Ci (ppm)
Control (0)	42.91^d^	40.91^de^	51.82^e^	40.59^d^	35.75^e^	10.32^e^	35.33^a^	16.64^d^	0.57^c^	115.74^e^	455.6^de^
20	41.25^d^	39.54^e^	49.01^e^	37.37^d^	38.19^e^	12.25^e^	24.28^b^	14.38^d^	0.52^c^	120.47^e^	378.3^e^
40	43.37^d^	45.29^de^	50.44^e^	40.92^cd^	41.95^de^	13.51^de^	20.48^b^	18.57^d^	0.73^b^	125.11^e^	470.4^d^
60	52.45 ^c^	48.97^d^	64.31^d^	50.82^c^	46.68^d^	16.25^d^	19.12^c^	20.33^cd^	0.96^ab^	144.86^d^	488.2^cd^
80	54.41 ^c^	58.58^c^	76.75^c^	60.84^bc^	52.26^c^	25.71^c^	18.59^c^	21.35^c^	1.01^a^	180.82^c^	502.4^c^
100	61.47 ^b^	68.62^b^	83.03^b^	70.21^b^	60.87^b^	33.45^b^	7.73^d^	30.22^b^	1.33^a^	203.68^b^	602.2^b^
200	77.44 ^a^	85.46^a^	97.18^a^	89.88^a^	76.66^a^	46.53^a^	4.92^d^	39.21^a^	1.38^a^	288.2^a^	1089.4^a^

Different letters denote significant differences (p ≤ 0.05) among ZnO NPs concentrations. RWC, relative water content; LAI, leaf area Index; MSI, membrane stability index; SDW= shoot dry weight; RDW= root dry weight; Pn, net photosynthesis; E, transpiration rate; gs, stomatal conductance and Ci, internal carbon dioxide concentration.

The increase in plant height and leaf area observed in response to the nanoparticles might be associated with nutritional behavior of particles or dissociated ions as well as the intricate function of Zn on crucial processes such as plant growth and development (Mukherjee et al., 2016). Studies have also highlighted that the increase in vegetative growth is linked to the role of zinc in controlling enzymes, protein synthesis, cell elongation, structural stability of cell membrane (Cakmak, 2008; Boonchuay et al., 2013) and speeding up metabolism (Singh et al., 2013). Overall, our results support the growth-promoting potential of ZnO NPs (Dimkpa et al., 2017; Neto et al., 2020) due to dissociated Zn^+2^ which can play a prominent role in the synthesis of tryptophan a precursor for the biosynthesis of auxin a plant growth hormone (Brennan, 2005; Faizan and Hayat, 2019; Srivastava et al., 2021). Furthermore, the observed improved mustard plant growth could emanate from the positive effect of ZnO NPs on photosynthesis (Xu et al., 2018).

### 3.3 Effects of ZnO NPs on physiological traits of *B. juncea*

**Figure 6:**
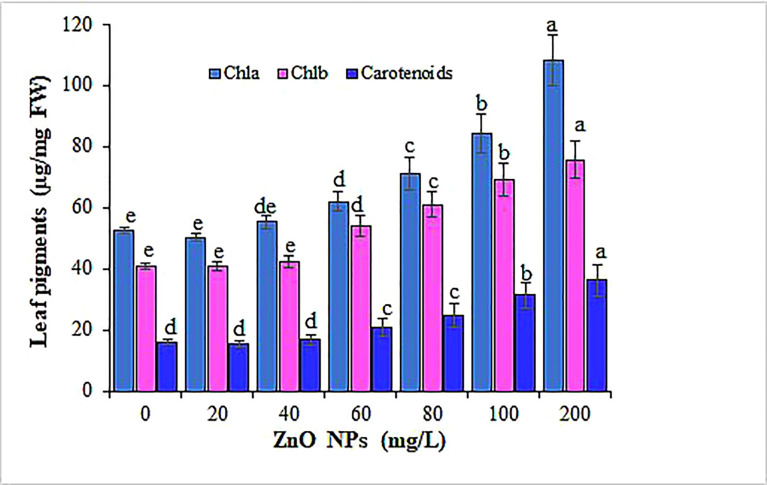
Variation in photosynthetic pigments content of mustard leaves under different concentrations of ZnO NPs. Values are mean standard error. Different letters denote significant differences (p ≤ 0.05) among concentrations for each pigment.

Several studies have shown that nanoparticle exposure significantly altered the total chlorophyll content and photosynthetic performance in various plants in concentration gradients (Baskar et al., 2018; Wang et al., 2018). Contrastingly, in our study, the treatment of mustard plants with ZnO NPs revealed an increase in the total chlorophyll content and their response was concentration-dependent (**Table 1**; **Figure 6**). The augment in the SPAD values significantly varied between concentrations (P < 0.05). Among the different tested concentrations of ZnO NPs, the application of 200 mg L^-1^ of ZnO NPs proved to be most effective and increased the SPAD by 53% over the control. Spectrometric analysis was applied to further examine the effect of ZnO NPs on the different photosynthetic pigments content (chlorophyll a, chlorophyll b, and carotenoid). The data showed an overall significant increase in chlorophyll a, chlorophyll b, and carotenoids (P < 0.05) in the mustard plant along with an increased ZnO NPs concentration than the control (**Figure 6**). The maximum increase in chlorophyll a, chlorophyll b, and carotenoids contents (51%, 46% and 56%, respectively) were recorded in the mustard plants treated with 200 mg L^−1^ ZnO NPs (*p* < 0.05). The present results corroborate with the findings of Prasad et al. (2012); Raliya et al. (2015); Venkatachalam et al. (2017); Samreen et al. (2017) and Narendhran et al. (2016) who studied the effect of ZnO NPs on the content of photosynthetic pigments in cotton, peanut, tomato, mung bean and sesame, respectively. This increase in photosynthetic pigments can be rationalized based on the contribution of zinc in chlorophyll synthesis and development and protochlorophyllide biosynthesis (Faizan and Hayat, 2019). Also, this might be due to metal nanoparticles being powerful amplifiers of photosynthetic effectiveness that in parallel can cause light absorption by chlorophyll, as it causes the transfer of energy from chlorophyll to nanoparticles (Mohsenzadeh and Moosavian, 2017). The addition of ZnO NPs leads to more nitrogen uptake and subtly stimulates nitrogen metabolism invaluable for chlorophyll molecules synthesis (Dimkpa et al., 2019). The underlining mechanism in the increased chlorophyll content as well could be linked to the role of Zn as a vital nutrient in the biosynthesis of chlorophyll (Faizan and Hayat, 2019). Furthermore, progress in the translation of chlorophyll biosynthetic genes, rate of chlorophyll aging, and associated proteins in the photosystem antenna complex could be ascribed to the rise in chlorophyll content with the addition of ZnO NPs (Bajguz and Asami, 2005; Sadeghi and Shekafandeh, 2014; Faizan et al., 2021). In contrast, Wang et al. (2016) have shown a decrease in the expression of chlorophyll biosynthesis and photosystem-associated genes that eventually reduced chlorophyll a and b in Arabidopsis plants that received ZnO NPs.

ZnO NPs amplify the photosynthetic efficiency by increasing the chlorophyll capacity to absorb light through energy transfer from ZnO NPs to chlorophyll molecules, in turn, triggers the boost in photosynthetic pigment contents (Faizan and Hayat, 2019). In the present study, consistent with an increase in chlorophyll content, measures of gas exchange parameters such as net photosynthetic rate (PN), stomatal conductance (gs), internal CO_2_ concentration (Ci), and transpiration rate (E) were increased significantly (P < 0.05) in mustard plants treated with ZnO NPs (**Table 1**). The mustard plants that received ZnO NPs (200 mg L^–1^) revealed the highest values of *Pn* (58%), *g*s (60%), *Ci* (58%), and *E* (59%) in comparison with control plants. In contrast to our results, Faizan and Hayat (2019) have reported the maximum decrease in photosynthetic parameters even at 200 ppm of ZnO NPs. The improvement in photosynthetic traits following the exposure to ZnO NPs may be due to the boost in light acquisition that further helps to shield the chloroplast from aging and eventually leads to enhanced photosynthesis (Yang et al., 2006; Xu et al., 2018). Metallic oxide nanoparticles can stimulate the net photosynthesis rate in photosynthetic systems either directly or indirectly affecting photosynthetic machinery in plants (Govorov and Carmeli, 2007). ZnO NPs improve stability and photosynthetic efficiency by enhancing antioxidant systems and boosting proline accumulation (Faizan et al., 2018). It is claimed that enhanced photosynthetic efficiency after the application of ZnO NPs could be caused by the improved activity of the water-splitting system during the light reaction, photochemical extinction, non-photosynthetic quenching, maximum PSII efficiency, and heightened rubisco activity (Yu et al., 2004; Siddiqui et al., 2018). High records of transpiration rate following exposure to ZnO NPs can be related to increased stomatal conductance (**Table 1**).

In addition to the effect of ZnO NPs on gas exchange measures, effects on membrane stability index (MSI) and leaf relative water content (RWC) were determined. Provision of 200 mg L^-1^ ZnO NPs overall increased MSI and RWC significantly by 55% and 47%, respectively, followed by 42% and 38% correspondingly with 100 mg L^-1^ (P < 0.05; **Table 1**) over the control. The increase in MSI could be explained by the fundamental roles of zinc in the maintenance of membranes integrity and in reducing the effect of lipid peroxidation due to the accumulation of reactive oxygen species (Bagci et al., 2007; Wang et al., 2018; Rai-Kalal and Jajoo, 2021; Noohpisheh et al., 2021). Similarly, the addition of ZnO NPs improved membrane stability and plant water status of eggplant (Semida et al., 2021). On the other hand, the augment in RWC might be associated with water potential adjustment owing to the increased uptake of water and macro- and micro-nutrients (Semida et al., 2021), particularly Fe, K, and Zn accumulation in the presence of ZnO NPs (**Table 2**). Furthermore, higher RWC in ZnO NPs treated mustard plants might be due to improved acquisition of osmolytes as supported by the increased MSI (Geremew et al., 2021).

**Table 2:** Effects of ZnO NPs application on macro- and micronutrients of *B. juncea* leaves.

Treatment (mg/L)	Macronutrients (ppm)				Micronutrients (ppm)					
	Ca	K	Mg	Na	P	B	Cu	Fe	Mn	Zn
Control (0)	25221 ± 11	21302.0 ± 47	2777.7 ± 11	7761.7 ± 9.21	12491.5 ± 2	53.1 ± 0.4	6.9 ± 0.2	65.4 ± 0.34	15.93 ± 0.1	58.5 ± 0.4
20	23054.9 ± 26	23769.7 ± 38.4	2842 ± 4.8	7665 ± 14.7	8787.8 ± 18	51.3 ± 0.4	5.2 ± 0.4	69.92 ± 0.66	15.7 ± 0.2	76.1 ± 0.2
40	22564.43+7	24474.6 ± 26.26	2470.7 ± 3.1	7336.75 ± 5.11	10008.1 ± 15	41.2± 0.2	4.6 ± 0.1	75.66 ± 0.66	10.4 ± 0.1	93.8 ± 0.3
60	21889.6 ± 26	25426.5 ± 44.3	2363.1 ± 4.9	6790.2 ± 10.88	9613 ± 21.74	47.6 ± 0.4	5.5 ± 0.1	88.1 ± 0.6	16.7 ± 0.3	101 ± 0.5
80	21810.4 ± 25	26684.6 ± 34.34	2576.71 ± 3	6529.2 ± 13.22	9139.1 ± 33	39.8 ± 0.2	4.8 ± 0.3	89.5 ± 0.4	11.56 ± 0.3	149.9 ± 0.3
100	21790.8 ± 25	30652.8 ± 26.26	2537.58 ± 7	5459.5 ± 4.99	9411.5 ± 17.4	41.1 ± 0.4	5.9 ± 0.3	115.3+0.9	13.04 ± 0.1	247.5 ± 1.3
200	18927 ± 19	32145.9 ± 54.54	2305.7 ± 3.7	4364 ± 7.1111	7549.6 ± 12.3	40.23 ± 0.1	3.9 ± 0.1	152.7 ± 0.88	11.7 ± 0.1	338.9 ± 0.4

Values are mean ± standard error.

### 3.4 Flavonoid content and total antioxidant capacity

**Figure 7:**
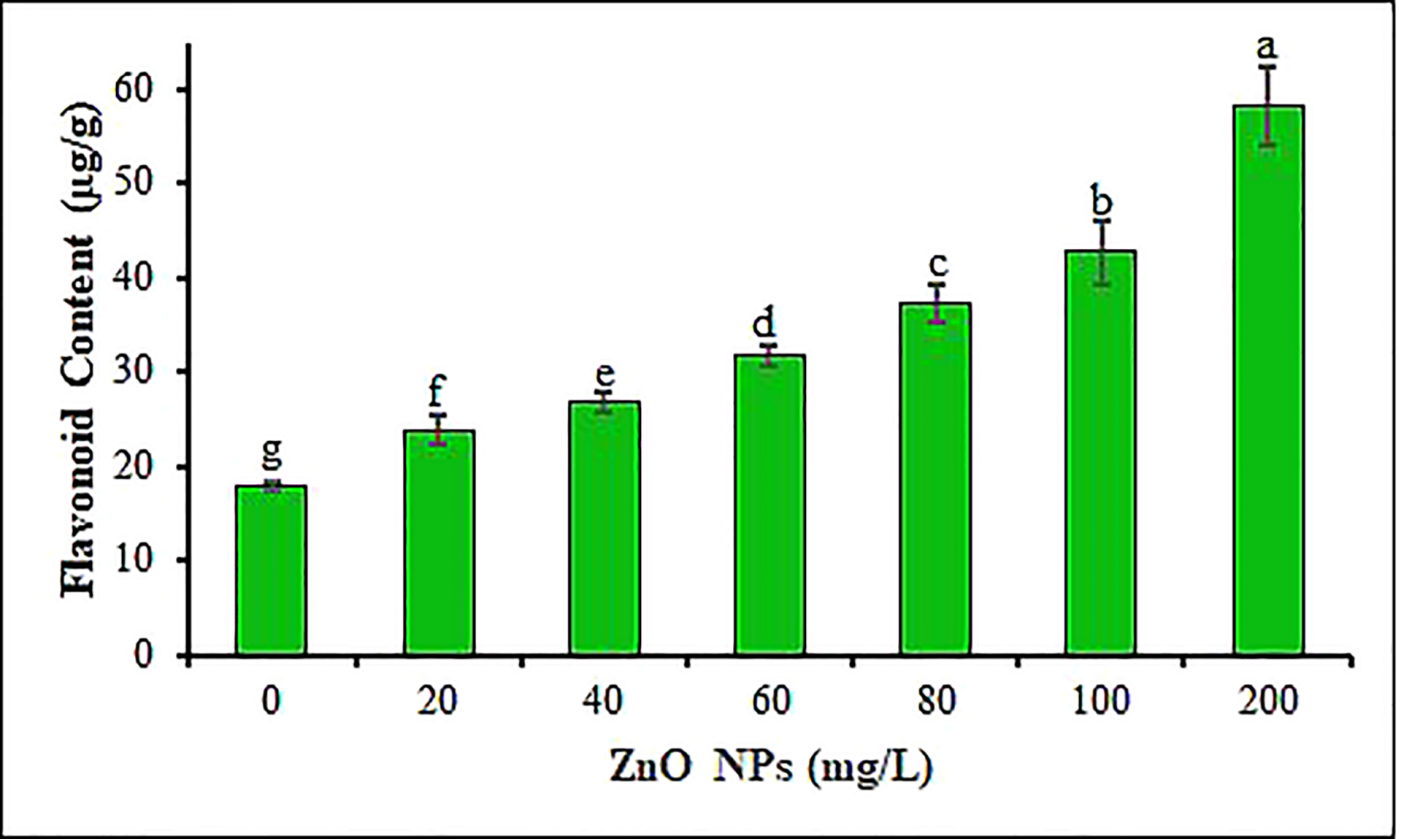
Total flavonoid contents of mustard leaves under different concentrations of ZnO NPs. Values are mean standard error. Different letters denote significant differences (p ≤ 0.05) in total flavonoid content among ZnO NPs concentrations.

Total flavonoid contents (TFC) in response to all six ZnO NPs concentrations are shown in **Figure 7**. This study indicated that the application of ZnO NPs significantly induced total flavonoid synthesis in *B. juncea* leaves in a concentration-dependent pattern compared to the control (P < 0.05). To overcome oxidative stress due to the metallic nanoparticles, plants activate their antioxidant defense system encompassing phenols and flavonoids which serve as metal chelators (through electron donation) and natural scavengers of ROS (Ilboudo et al., 2012; Gupta and Pandey, 2020; Hussain et al., 2021). In the current study, maximum TFC in mustard leaves (58 µg g^-1^) was recorded at the 200 mg L^-1^ ZnO NPs treatment followed by a 100 mg L^-1^ with TFC of 43 µg g^-1^ (**Figure 7**). In support of our results, Zafar et al. (2016) have also reported increase in TFC in one of the closely related species of *B. juncea*, namely *B. nigra* seedlings treated with ZnO NPs. Comparably, the ZnO NPs treatment was shown to increase the contents of TFC in *Glycyrrhiza glabra* seedlings (Oloumi et al., 2015), *Persicaria hydropiper* (Hussain et al., 2021) *Raphanus sativus* (Mahmoud et al., 2019) and *Vicia faba* (Mogazy and Hanafy, 2022) plants. The use of nanoparticles as oxidative stress producers resulted in the production of secondary metabolites like flavonoids and phenols that function as ROS scavengers (Javed et al., 2017; Vela´zquez-Gamboa et al., 2021; Mogazy and Hanafy, 2022).

**Figure 8:**
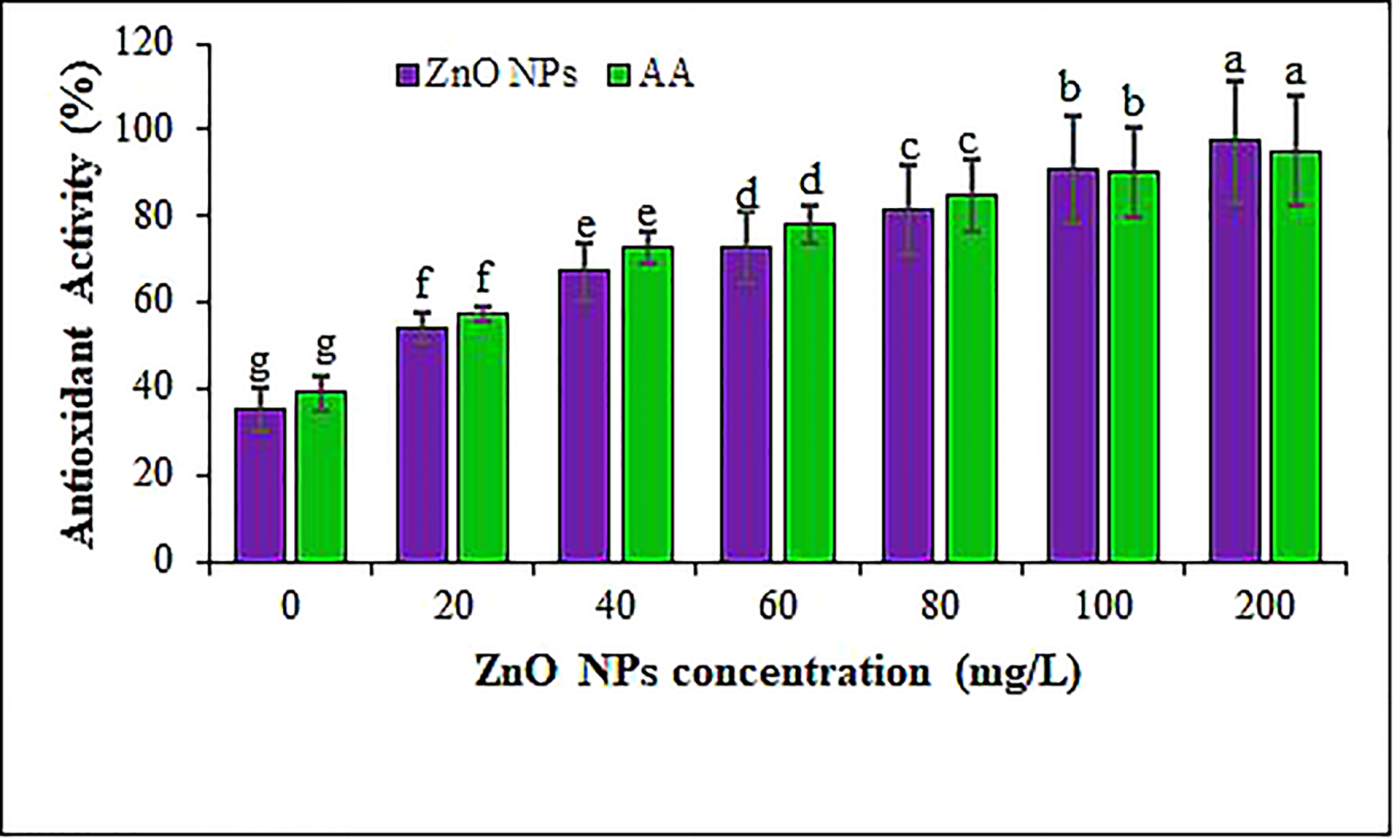
The antioxidant activity of ZnO NPs measured using DPPH radical scavenging assay. AA indicates ascorbic acid. Different letters denote significant differences (p ≤ 0.05) across different concentrations for a particular antioxidant. Values are mean standard error.

The antioxidant potential was measured using DPPH free radical scavenging assay in the *B. juncea* leaves under the different ZnO NPs treatments. Amendment of *B. juncea* plants using ZnO NPs revealed that antioxidant activity in leaves was significantly boosted from 54% (in 20 mg L^-1^ of ZnO NPs) to 97% (in 200 mg L^-1^ of ZnO NPs) relative to the control (35%) (**Figure 8**). These results corroborated previous findings (Ushahra et al., 2014; Singh et al., 2018; Iziy et al., 2019) that reported a positive impact of ZnO NPs on antioxidant activities of different plant species. The antioxidant activity in the mustard leaves of plants provided with different concentrations of biosynthesized ZnO NPs confirms that the addition of ZnO NPs promotes the biosynthesis of compounds with antioxidant activity like flavonoids and phenols. Nevertheless, the augment or decline of antioxidant activity is a function of the balance between the antioxidant activity of metabolites and the degree of oxidative stress (Baskar et al., 2018; Srivastava et al., 2021). On the other hand, our results contradict the findings of Javed et al. (2017) who studied the effect of ZnO NPs on the TFC of *Stevia rebaudiana* and demonstrated a reduction in the TFC of the plants treated with 100 and 1,000 mg L^−1^ of ZnO NPs, compared to the control plants. Zhu et al. (2013) highlighted that the antioxidant capacity could be affected by the levels of metals like zinc, which function as cofactors for enzymes, signaling molecules and transcription factors. Overall, the application of ZnO NPs resulted in the enrichment of Zn^2+^ which could increase the nutritional quality and antioxidant activity for human consumption as a functional food.

### 3.5 Macro and micro-nutrient content

An ICP-OES analysis was carried out to measure the amount of P, K, Ca, B, Cu, Mg, Fe, Mn, Na, and Zn content in ZnO NPs exposed mustard plant leaf samples. The application of ZnO NPs did significantly affect the mustard green concentration of some essential and beneficial nutrients both positively and negatively including Zn (**Table 2**). The application of ZnO NPs had significantly increased the K, Fe and Zn accumulation and in contrast, significantly decreased Ca and Na content following dose-dependent (P < 0.05). Besides, the ZnO NPs had no significant effect on Mg, P, Cu, B and Mn (P > 0.05). The K, Fe and Zn accumulation in plants subjected to 200 mg L^-1^ ZnO NPs was 34%, 17% and 83% higher than that of the control group, respectively. Compared with the control, the content of Ca and Na significantly decreased by 33% and 67%, respectively, in mustard plants exposed to the highest concentration of the nanoparticles (200 mg L^-1^). The lowest P content was observed under the highest ZnO NPs concentration (200 mg L^-1^). This could be due to the toxic effect of the nanoparticles on P-solubilizing microorganisms and decreased enzyme activity and consequently impacting P uptake of plants (Chai et al., 2015; Raliya et al., 2016). In agreement with the present study, the application of ZnO NPs increased K, Fe and Zn in different plants (Dimkpa et al., 2019; Grangah et al., 2020; Semida et al., 2021). Therefore, amendment with Zn ONPs improved the nutritional status of *B. juncea*.

Though the mechanisms of how ZnO NPs affect the content of other nutrients have not been clearly established Haynes (1980) and Dimkpa et al. (2019) suggested that divalent metal ions (Zn^2+^) in the root change potential across the cell membrane thus enabling the uptake of monovalent cations such as K. Alternatively, the influence of Zn from the nanoparticles on the content of particular nutrients may be linked to synergistic or antagonistic interactions and which varies strongly with nutrient ratios (Dimkpa et al., 2017; Rietra et al., 2017; Dimkpa et al., 2019; Geremew et al., 2021).

### 3.6 Zn accumulation and ROS in mustard leaves

**Figure 9:**
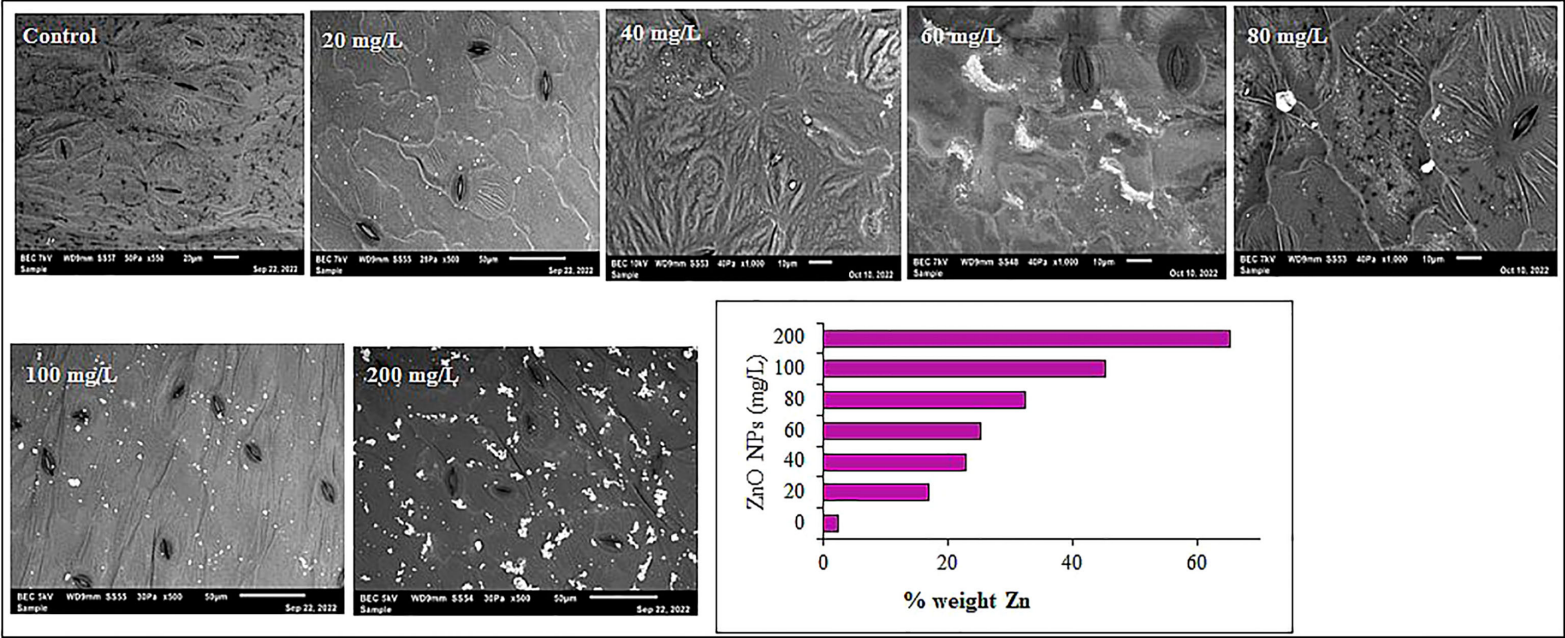
SEM analysis showing Zn accumulation in mustard leaves from plants treated with different ZnO NPs concentrations. The bar plot from EDX shows the percent weight of Zn detected from each leaf.

Mustard leaves were examined under SEM to detect the bioaccumulation of ZnO NPs in the leaves. In support of the ICP analysis, the SEM coupled with the EDX analysis revealed that Zn content in *B. juncea* leaf increased with a dose of ZnO NPs supplemented from 3% to 65% weight (**Figure 9**). However, the amounts of Zn detected at the low concentrations of nanoparticles were not significant (P > 0.05). On the other hand, the maximum Zn accumulation (65%) was observed in 200 mg L^-1^ ZnO NPs treated mustard leaves. The aggregation of ZnO NPs in the leaves was evident in several spots of Zn in the SEM images. The high accumulation of Zn has also been reported in plant tissues by various recent findings performed with Zn-based NPs (Singh and Kumar, 2016; Zoufan et al., 2020; Alsuwayyid et al., 2022). The SEM images also provide supporting evidence on how ZnO NPs can improve the nutritional status or zinc content of mustard plants by strengthening the vascular system (Pejam et al., 2021) and enhancing nutrient uptake efficiency by regulating nanoscale plant pores (Abd El-Aziz et al., 2019; Srivastava et al., 2021).

Considering the discrepancy in magnitude of Zn content between the ICP and SEM analyses as well as the size of the ZnO NPs used (84.5 nm), the dissolution of ZnO NPs to preferentially absorbed Zn ions or directly adsorbed ZnO NPs could result in the higher Zn content in plant leaves (Xu et al., 2018; Pejam et al., 2020). Despite the mechanism of absorption and translocation of nanoparticles from the soil to the different plant tissues are still the subject of research. The absorption and translocation of metallic and metallic oxide nanoparticles counterparts occur in a similar way as micro and micronutrients (Fraceto et al., 2016). It has been thought that nanoparticles are assimilated by the root hairs and further proceed through cellular pores following either the symplastic or apoplastic or a combination of both pathways (Rico et al., 2011; Lambreva et al., 2015; Rajput et al., 2018; Usman et al., 2020). It is apparent from recent studies that Zn may be accumulated in plant tissues and cellular and sub-cellular organelles and regulate cellular organizations (Bradfield et al., 2017; Wang et al., 2018; Radi et al., 2018). The uptake of NPs by several plants led to their accretion in subcellular locations (Schwab et al., 2016; Faizan et al., 2018), Overall, the uptake of the nanoparticles relies on the plant anatomy and shape, composition and size of NPs (Wang et al., 2018).

**Figure 10:**
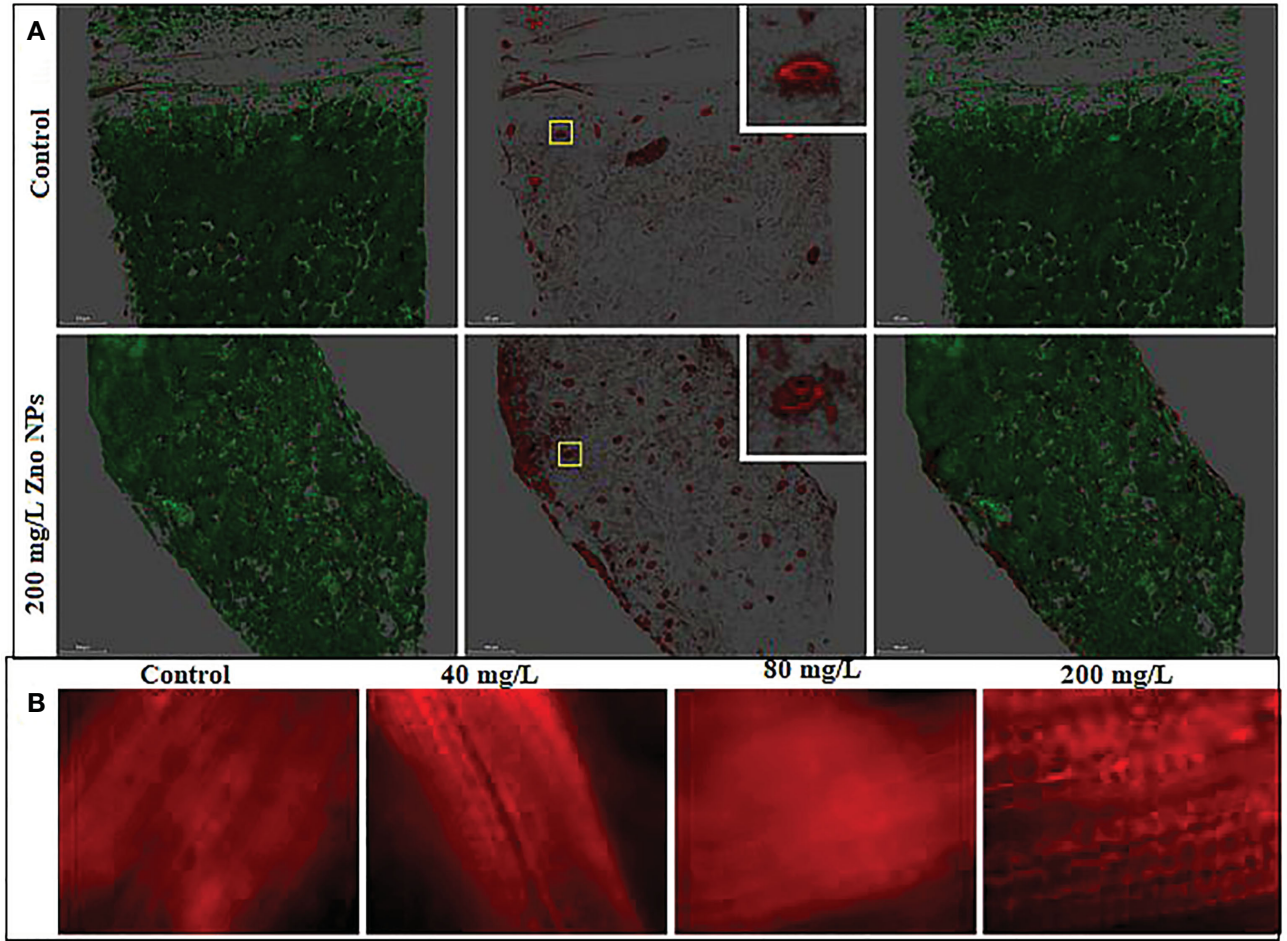
Increase in reactive oxygen species (superoxide) in mustard leaves treated with ZnO NPs and stained with Mitosox. **(A)** Confocal images of the structure of chloroplast and stomata. The boxed region represents the magnified region. **(B)** Bright field fluorescent images of the leaf veins. The intensity of the dye represents the high accumulation of superoxide in the leaf veins.

The major forms of reactive oxygen species (ROS) includes hydrogen peroxide (H_2_O_2_), superoxide (O_2_^.-^_)_, singlet oxygen (^1^O_2_) and the hydroxyl radical (HO^-^), mainly produced in the chloroplast, mitochondria and peroxisomes during environmental stress in plants (Das and Roychoudhury, 2014; Faizan et al., 2021). ROS signaling from plant stomata plays a critical role in innate immunity and defense mechanism (Castro et al., 2021; Mittler et al., 2022). In stomatal closure, NADPH oxidase catalyzes the transfer of electrons from NADPH to ^1^O_2_ form O_2_^.-^, then to H_2_O_2_ (Sierla et al., 2016). Mitosox staining for superoxide showed that ROS are accumulated in stomata (**Figure 10A**). When treated with 200 mg L^-1^, the level of ROS is slightly enhanced. ROS is also accumulated on the leaf midrib and minor lamina veins (**Figure 10B**). Fluorescent microscopy images showed the ROS in the leaf vein coil structure of plants treated with ZnO NPs. However, their effect on the disruption of physiological processes such as chlorophyll content and photosynthesis were not observed. Like other plant species, *B*. *juncea* might overcome such effects of ROS with an intricate non-enzymatic and enzymatic antioxidant system (Ali et al., 2008; Faizan et al., 2018; Faizan and Hayat, 2019). According to Faizan and Hayat (2019) exogenous application of ZnO NPs elevated the enzymatic defense mechanisms by increasing the synthesis of catalase, peroxidase and superoxide dismutase. On the other hand, zinc ions from the ZnO NPs benefit in raising the expression of antioxidant genes in plants by supporting non-enzymatic antioxidant production and eventually overcoming the influence of ROS (Hassan et al., 2020; Adhikari et al., 2020; Aazami et al., 2021). Phenols and flavonoid accumulation recorded in this study is part of the adaptive response acting as ROS scavengers either in conjunction with or individually of antioxidative enzymes (Mogazy and Hanafy, 2022). Despite the accumulation of Zn in plant tissue that results in ROS increase, the enhanced production of flavonoids with radical scavenging potential, overall, in this current study indicated that no ZnO NPs toxicity was noted in the mustard plants in terms of growth and physiological performance. This suggests that ZnO NPs stimulate ROS signaling to enhance the defense mechanism in mustard plants.

## 4 Conclusions

This study highlights the effect of biosynthesized ZnO-NPs using pecan leaves at different concentrations on *B. juncea* (mustard) plant growth, chlorophyll content, relative water contents, membrane stability, and net photosynthesis rate. Application of ZnO NPs up to 200 mg L^-1^ enhances nutrient accumulation including Zn, Fe and K, flavonoids and antioxidant potentials in mustard leaves and then reduces the effect of ROS. Therefore, ZnO NPs can be potentially used as a plant growth stimulant and as a novel soil amendment for enhancing crop yields. Besides, the biofortification of *B. juncea* plants with ZnO NPs helps to improve the nutritional quality of the crop and perhaps potentiates its pharmaceutical effects. Moreover, further investigations are required to examine the effect of ZnO NPs on different secondary metabolites and the mechanisms at a molecular level for extensible applications as nanofertilizers and the synthesis of nanocomposites.

## Data availability statement

The raw data supporting the conclusions of this article will be made available by the authors, without undue reservation.

## Author contributions

AG developed the concept including the methods, data curation, formal statistical analysis, and writing of the original draft. LC contributed to resources, funding acquisition, project administration, evaluation of the experimental approach, assisted in the development of the project idea and the review of the manuscript. SW conducted ICP-OES analysis of nutrients and review. HW performed the ROS analysis. NB and SR carried out the XRD analysis and verification of the data as well as a review of the manuscript. EP gathered data related to RWC, biomass and LA as well as participated in the synthesis of ZnO NPs. PS provided the facility to carry out inflorescence analysis of ROS. AW verify the nomenclature of the plants used for the synthesis of ZnO NPs as well as reviewed the manuscript. All authors contributed to the article and approved the submitted version.

## Supplementary material

Supplementary Figure 1 Summary of measured parameters and measurement times after germination.

Supplementary Figure 2 Phytotoxicity test of ZnO NPs on mustard seeds germination **(A)**, partial view of seedlings vary in height **(B)** and percent germination **(C)**.
