# Supplementary figures and images for: Effect of zinc oxide nanoparticles synthesized from Carya illinoinensis leaf extract on growth and antioxidant properties of mustard (Brassica juncea)
Source: Front Plant Sci. 2023 Jan 23;14:1108186. doi: 10.3389/fpls.2023.1108186 (PMC9900026; doi:10.3389/fpls.2023.1108186)

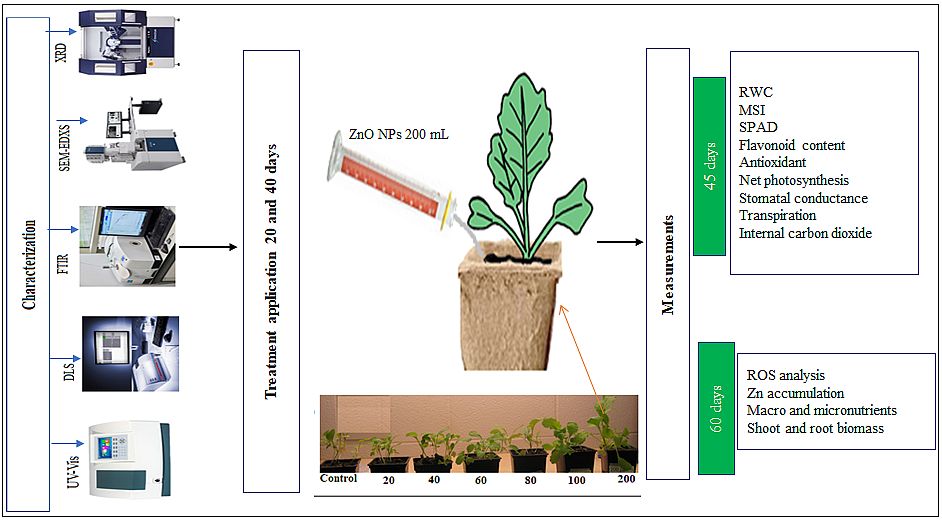


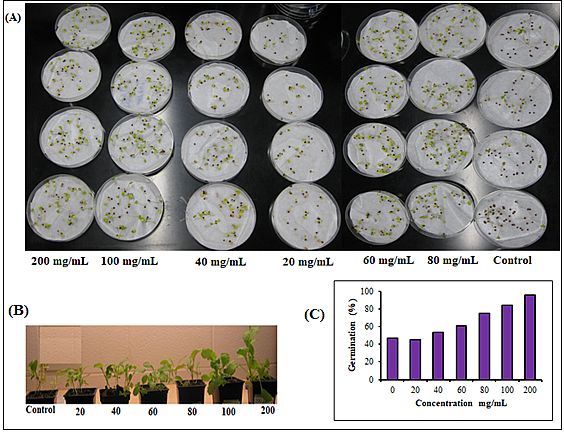

Supplement: Supplementary Figure 1 — Summary of measured parameters and measurement times after germination. [file DataSheet_1.docx]
